# The Drag Crisis Phenomenon on an Elite Road Cyclist—A Preliminary Numerical Simulations Analysis in the Aero Position at Different Speeds

**DOI:** 10.3390/ijerph17145003

**Published:** 2020-07-11

**Authors:** Pedro Forte, Jorge E. Morais, Henrique P. Neiva, Tiago M. Barbosa, Daniel A. Marinho

**Affiliations:** 1 Department of Sports, Douro Higher Institute of Educational Sciences, 4560-708 Penafiel, Portugal; morais.jorgestrela@gmail.com; 2 Department of Sports Sciences and Physical Education, Instituto Politécnico de Bragança, 5300-253 Bragança, Portugal; barbosa@ipb.pt; 3 Research Center in Sports, Health and Human Development, CIDESD, 6201-001 Covilhã, Portugal; henriquepn@gmail.com (H.P.N.); marinho.d@gmail.com (D.A.M.); 4 Department of Sports Science, Beira Interior University (UBI), 6201-001 Covilhã, Portugal

**Keywords:** cycling, drag coefficient, drag crisis, numerical simulations

## Abstract

The drag crisis phenomenon is the drop of drag coefficient (*C_d_*) with increasing Reynolds number (*Re*) or speed. The aim of this study was to assess the hypothetical drag crisis phenomenon in a sports setting, assessing it in a bicycle–cyclist system. A male elite-level cyclist was recruited for this research and his competition bicycle, helmet, suit, and shoes were used. A three-dimensional (3D) geometry was obtained with a 3D scan with the subject in a static aero position. A domain with 7 m of length, 2.5 m of width and 2.5 m of height was created around the cyclist. The domain was meshed with 42 million elements. Numerical simulations by computer fluid dynamics (CFD) fluent numerical code were conducted at speeds between 1 m/s and 22 m/s, with increments of 1 m/s. The drag coefficient ranged between 0.60 and 0.95 across different speeds and *Re*. The highest value was observed at 2 m/s (*C_d_* = 0.95) and *Re* of 3.21 × 10^5^, whereas the lower *C_d_* was noted at 9 m/s (*C_d_* = 0.60) and 9.63 × 10^5^. A drag crisis was noted between 3 m/s and 9 m/s. Pressure *C_d_* ranged from 0.35 to 0.52 and the lowest value was observed at 3 m/s and the highest at 2 m/s. The viscous drag coefficient ranged between 0.15 and 0.43 and presented a trend decreasing from 4 m/s to 22 m/s. Coaches, cyclists, researchers, and support staff must consider that *C_d_* varies with speed and *Re*, and the bicycle–cyclist dimensions, shape, or form may affect drag and performance estimations. As a conclusion, this preliminary work noted a drag crisis between 3 m/s and 9 m/s in a cyclist in the aero position.

## 1. Introduction

Athletes aim to save any time they are able to in time-based sports such as cycling. To improve their winning time, practitioners can employ a comprehensive set of techniques and methodologies [1,2]. Performance in time-based sports depends on speed [3]. In cycling, speed is the balance between mechanical power to overcome resistive forces and the mechanical power delivered [4]. In this sport it is possible to identify two main resistive forces: the drag and rolling resistance [4]. Rolling resistance is mainly dependent on the mass of the bicycle–cyclist system, speed, and rolling resistance coefficient, whereas drag is dependent on speed, drag coefficient, fluid density, and surface area. The mean speed of professional road cyclists during a stage is about 40 km/h (≈11 m/s) [5].

Drag accounts for 90% and rolling resistance for about 10% of the total resistive forces [6]. It is possible to assess resistive forces by experimental tests, analytical procedures and numerical simulations [2]. Numerical simulations by computational fluid dynamics (CFD) enable the assessment of aerodynamics in highly-controlled conditions. They show good adherence to experimental data from wind tunnel testing [2,7,8]. Moreover, CFD enables the breakdown of the total drag force into its components (pressure and viscous drag) [7,8,9,10,11,12].

The variable selected most often to assess aerodynamics is the effective surface area (product of frontal surface by drag coefficient, *C_d_*) [4,8,9]. However, some analysts used to assume the drag coefficient (*C_d_*) was invariant at different speeds [13,14]. Notwithstanding, variations in effective surface area have been explained by a phenomenon known as drag crisis. The drag crisis is a drop in *C_d_* at different *Re* and speeds due to a shift in the fluid flow behavior [9,15]. The fluid flow behavior around a body affects the *C_d_* [8,16]. A low flow speed, of about *Re* = 2 × 10^5^, is called a sub-critical regime [17]. A high *Re* value is known as a critical regime [18]. The super-critical regime is characterized by the transition from the laminar to turbulent flow. In a super-critical regime the *Re* is higher than in a critical regime [17,18,19]. The drag crisis has been reported in cycling [20] and spheres [15,21,22] and seems to occur at *Re* numbers between 4 × 10^5^ and 8 × 10^5^ [15]. The *Re* when that drag crisis occurs is called critical *Re* [15,20]. It was reported that for smooth spheres, the *C_d_* drops, reaching a bottom at the end of the critical regime (i.e., 3.5 × 10^5^ < Re < 5 × 10^5^). Then, *C_d_* seems to rise at around Re = 6.5 × 10^5^ and Re = 10^6^ [19]. The transition from a sub-critical to super-critical regime encompasses the transition from laminar to turbulent flow regime [15,20]. This transition is characterized by high pressure and flow velocity variations, whereas laminar flow is characterized by the non-disruption of fluid flow layers [15].

As far as the authors’ understanding goes, only two papers have assessed the drag crisis in cycling, notably on a cyclist’s leg [20] and the wake length influence on *Re* [23]. However, no study has reported the *C_d_* variations across different speeds on an elite road cyclist. Most studies assessing the drag crisis are conducted with smooth spheres, balls, and aerofoils [15,21,22]. No study was found with more complex geometries such as a bicycle–cyclist system. Little evidence on such a phenomenon is found in sports. Indeed, the drag crisis might occur in different bodies in sports settings just as in a bicycle–cyclist system. Forte et al. [24] suggested assessing *C_d_* across different speeds, even though, at least in sports sciences, analysts and researchers are prone to assume that *C_d_* is kept constant across a range of speeds [25].

It is a standard procedure to test cyclists in the aero position [24,25]. However, it is yet unclear if the phenomenon under appreciation is present when testing cyclists in the aero position. If so, it can explain, at least to some extent, drag variations as noted beforehand [24,25,26]. A deeper insight into the drag crisis and its effect on drag force can also be an aid to end-users. It can help end-users (cyclists, coaches, analysists, etc.) to run more accurate aerodynamics and performance forecasts.

The aim of this study was to assess the hypothetical drag crisis phenomenon in a sports setting, assessing it in a bicycle–cyclist system. It was hypothesized that a drag crisis phenomenon would be observed in a bicycle–cyclist system.

## 2. Materials and Methods

### 2.1. Subject

A male elite level cyclist was recruited for this research. The subject participated in national competitions, weighed 65.00 kg and was 1.76 m tall; the bicycle weighed 7 kg. The subject’s height was measured with the participant looking forward, shoulders relaxed, arms at sides, legs straight and knees together, the feet flat and heels almost together. The participant had an upper leg length of 0.60 m, shoulder/biacromial breadth of 0.39 m, biiliac breadth of 0.32 m, upper arm length of 0.36 m, abdominal circumference of 0.76 m, arm circumference 0.32 m, hip circumference 0.98 m, and thigh circumference 0.54 m. The skin folds were not measured. All procedures were in accordance with the Helsinki Declaration regarding human research and informed written consent by the volunteer subject was obtained beforehand. The scientific committee of the Higher Institute of Educational Sciences of the Douro approved the research (PROJ1.576).

### 2.2. Scanning the Model

**Figure 1:**
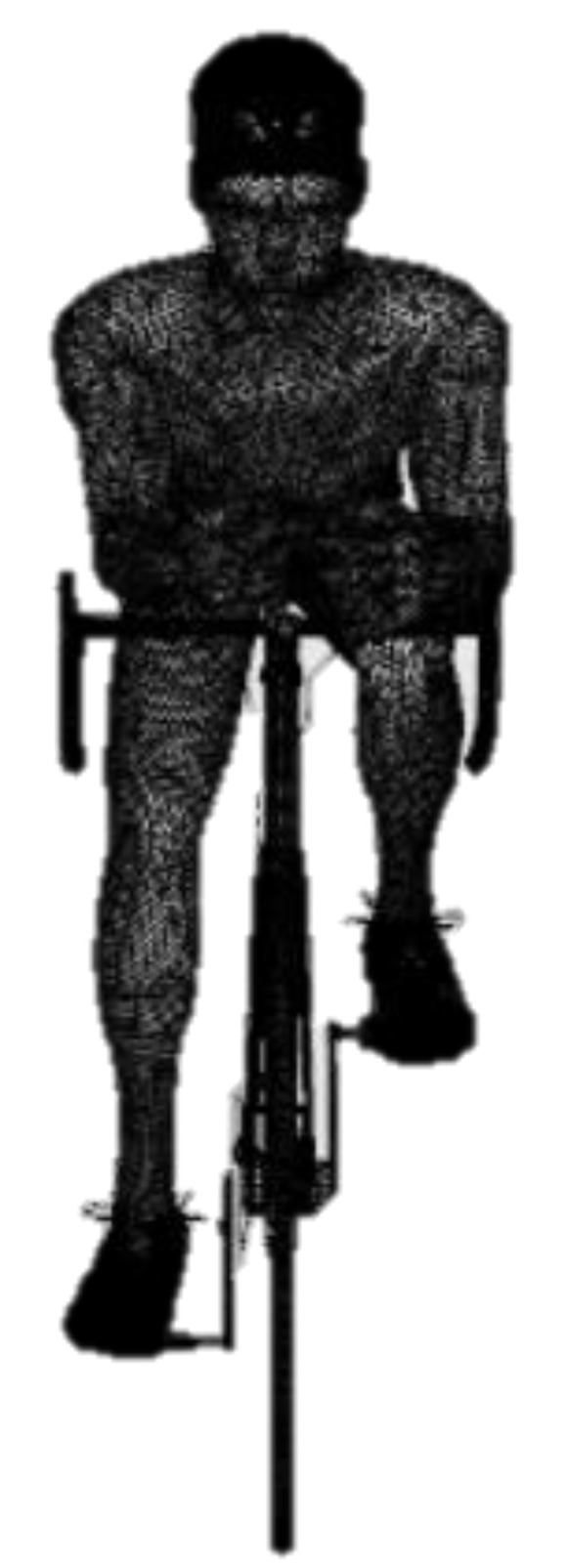
The meshed geometry in the aero position.

The subject and bicycle were scanned in the aero position (Figure 1). The cyclist wore racing clothes, shoes and helmet, and used his own bicycle. The subject was asked to maintain a static position during scanning. The bicycle was placed and fixed on a roller.

The scans were collected by a portable Sense 3D scanner (3D Systems, Inc., Rock Hill, SC, USA) and saved in the Sense Software (Sense, 3D Systems, Inc., Rock Hill, SC, USA). The scanner precision was 0.0009 m (0.9 mm) at 0.5 m (50 cm) distance. The Sense software allowed us to clean, fill holes and solidify the entire bicycle–cyclist geometry, then the model was exported as a stereolithographic file (.stl) [27]. The CAD models were created in Geomagic studio software (3D Systems, Rock Hill, SC, USA) [9].

### 2.3. Boundary Conditions

The Ansys Workbench geometry module software (Ansys Fluent 16.0, Ansys Inc., Canonsburg, PA, USA) enabled us to create a three-dimensional domain (length = 7 m; width = 2.5 m; height = 2.5 m) around the cyclist. The domain was meshed with more than 42 million elements to represent the fluid. The elements were prismatic and tetrahedral with cell size near 25.72 µm. The cyclist geometry was at 2.5 m from the inlet portion for each simulation [28].

Typically, a professional road cyclist reaches mean speeds of about 11 m/s (≈ 40 km/h) during a stage [5]. Thus, the numerical simulations were conducted between 1 and 22 m/s with increments of 1 m/s (22 speeds). The speeds were set at the inlet portion of the enclosure (-z direction). The turbulence intensity was assumed as 1 × 10^−6^% for different positions. The non-slip wall and scalable wall functions were assigned.

### 2.4. Numerical Simulations

The Reynolds-averaged Navier–Stokes (RANS) equations in fluent CFD code are solved by the finite volumes approach method. To solve these equations a turbulence model is required; the realizable k-ε was selected and the SIMPLE algorithm was used to solve the correction in velocities and pressure and satisfy the discrete continuity equation of the fluid flow behavior. The governing equations of the discretization schemes were defined as second order and the gradients were computed by the least-squares cell-based method. Pressure and momentum were set as second order and second order upwind. The turbulent kinetic energy and dissipation rate were defined as first order upwind [12]. The convergence occurred automatically in the Ansys Fluent 16.0 [24,25,26,27].

### 2.5. Outcomes

#### 2.5.1. Drag Force

The numerical simulations allows us to obtain the drag and the drag coefficient from Ansys Fluent Software [24,25,26,27]. For the drag force, Equation (1) was used:

(1) $$F_{d}=\frac{1}{2}\rho ACdv^{2}$$

where *F_d_* is the drag force, *C_d_* represents the drag coefficient, *v* the velocity, *A* the surface area and *ρ* is the air density (1.292 kg/m^3^) [24,25,26,27]. *C_d_* is given by Equation (2):

(2) $$C_{d}=\frac{\frac{1}{2}pAv^{2}}{F_{d}}$$

#### 2.5.2. Reynolds Number

Reynolds number was calculated as:

(3) $$R_{e}=\frac{pvL}{µ}$$

where *L* is the characteristic linear dimension and µ is the dynamic viscosity of the fluid (assumed as 0.00001813 Pa/s).

## 3. Results

**Figure 2:**
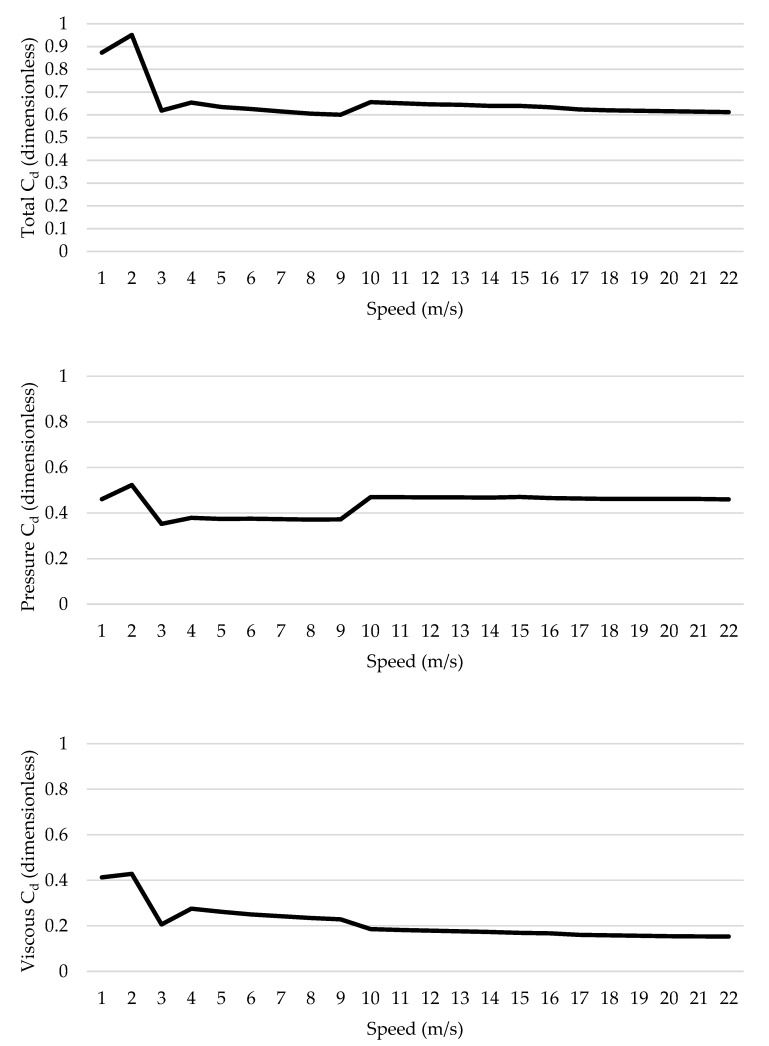
Drag coefficient (*C_d_*) variations across different speeds (**top panel**), pressure drag coefficient (**middle panel**) and viscous drag coefficient (**bottom panel**) across different speeds.

Drag coefficient variations are presented across different speeds and *Re* in Figure 2 (top panel). The highest value was observed at 2 m/s (*C_d_* = 0.95) with a *Re* of 3.21 × 10^5^. The lower *C_d_* was noted at 9 m/s (*C_d_* = 0.60) and 9.63 × 10^5^. The partial difference between lowest and highest *C_d_* was 37%. The *C_d_* seems to increase from 1 to 2 m/s (*Re* of 1.07 × 10^5^ to 2.14 × 10^5^) and drops to 3 m/s (*Re* = 3.21 × 10^5^). It is then possible to identify another *C_d_* decay between 4 m/s (*Re* = 4.28 × 10^5^) and 9 m/s (*Re* = 9.63 × 10^5^). Afterwards, *C_d_* increases from 9 m/s (*Re* = 9.63 × 10^5^) to 10 m/s (*Re* = 1.07 × 10^6^) and decreased from 10 m/s (*Re* = 1.07 × 10^6^) to 22 m/s (*Re* = 2.35 × 10^6^). Thus, a drag crisis between 3 m/s and 9 m/s in the bicycle–cyclist system was noted.

Pressure *C_d_* ranged from 0.35 to 0.52 and (Figure 2, middle panel). The partial change between lowest and highest values was 33% (Figure 2, middle panel). The lowest value was observed at 3 m/s and the highest at 2 m/s. Pressure *C_d_* seemed to increase from 1 m/s to 2 m/s and then decreased at 3 m/s. Pressure *C_d_* had variations up to 7%, between 3 and 9 m/s. From 9 m/s to 22 m/s, the pressure *C_d_* increased from 19% to 21%. Altogether, a crisis phenomenon was also noted in the pressure *C_d_* between 3 m/s and 9 m/s.

Viscous *C_d_* values ranged between 0.15 and 0.43, and the partial variation was 65% (Figure 2 bottom panel). A trend for viscous *C_d_* to increase from 1 m/s to 2 m/s and then decrease to 3 m/s was observed. From 4 m/s onwards, viscous *C_d_* decreased between 5% and 44%. Therefore, the drag crisis did not seem to be as obvious as in pressure *C_d_* and it can be argued that it had less influence on the total *C_d_* drop.

## 4. Discussion

The aim of this study was to assess the hypothetical drag crisis phenomenon in a sports setting, assessing it in a bicycle–cyclist system. It was verified that total, pressure and viscous *C_d_* differed across the different speeds. A *C_d_* drop was noted from 3 m/s to 9 m/s and from *Re* = 3.21 × 10^5^ to 9.63 × 10^5^.

In the current study, *C_d_* ranged between 0.60 and 0.95, from 1 m/s to 22 m/s, and it varied between 0.3% and 35%. From 1 m/s to 3 m/s, the *C_d_* increased and decreased over the selected speed range. A decreasing trend was observed from 3 m/s to 9 m/s, and between 10 m/s and 22 m/s the *C_d_* presented a trend to decrease with speed. Altogether, the drag crisis was observed between 3 m/s and 9 m/s and *C_d_* stabilized for speeds above 10 m/s. The *C_d_* depends on the fluid flowing around the body [8,16] and the fluid flow is dependent on the body’s geometry or shape [8,16]. Moreover, the drop in *C_d_* values can be explained by the fluid flow transition from laminar to turbulent. The fluid flow is characterized as sub-critical in laminar flows [15,20]. In this regime, the *C_d_* seems not to be stable, and may explain the variations between 1 m/s and 3 m/s due to the fluid layers’ viscosity breakup [15]. The dragged fluid on the body surface disrupts due to the speed and turbulence and, after the critical regime, it is expected that *C_d_* reaches the bottom value [19]. The critical regime seems to end at *Re* between 3.5 × 10^5^ and 5 × 10^5^ [19]. However, in the current study, the lowest *C_d_* = 0.60 was achieved at *Re* = 9.63 × 10^5^. The fluid flow transitions have been evaluated in spheres or airfoils [15,21,22]. An analysis with soccer ball aerodynamics revealed that drag crisis occurs near 8 m/s to 18 m/s and *C_d_* drops from 0.5 to 0.15 [29]. Yand and Cheng [30] performed a hydrodynamic analysis of a cylinder drag crisis with an O-tube and revealed that *C_d_* reduced from 0.9 to 0.35 at Re between 1.9 × 10^5^ and 2.7 × 10^5^. Conversely, in more complex geometries such as a bicycle–cyclist system, it was yet unclear if the same phenomenon would be present. For a cyclist in the time trial position, a position similar to the aero position, the *C_d_* is 0.60 at *Re* = 7 × 10^5^ [31,32]. In this study, *C_d_* was near 0.60 at 7.49 × 10^5^ < *Re* < 9.63 × 10^5^.

The total *C_d_* is given by the sum of pressure and viscous *C_d_*. Pressure *C_d_* varied from 0.35 and 0.52, and viscous *C_d_* between 0.15 and 0.43. The pressure *C_d_* partial contribution to total *C_d_* was between 53% and 75%; whereas, viscous *C_d_* was between 25% to 47%. The partial contribution of pressure *C_d_* increased with speed and, conversely, viscous *C_d_* contribution decreased with speed. This seems to be in agreement with the literature. Authors have reported that pressure drag is the main force responsible for aerodynamic resistance [26,28,31,32,33]. In the current study, the drag crisis phenomenon was noticeable in pressure *C_d_* between 3 m/s and 9 m/s and *Re* 3.21 × 10^5^ and 9.63 × 10^5^. This phenomenon seems to be related to the flow regimes transition (critical regime) from laminar to turbulent flow [15,19]. In the present study, it seems that the flow transitions happened between 3 m/s and 9 m/s. The viscous *C_d_* mainly decreased with speed. Therefore, based on these findings it seems that viscous *C_d_* is not responsible for the drag crisis phenomenon. Viscous drag encompasses the interaction between the body’s surface and the fluid [15]. Thus, due to the fluid flow transitions, viscous drag had a small contribution in comparison to pressure drag, as speed increases [9,15]. In the current research, the fluid (air) was dragged by sublayers to the bicycle–cyclist system surface. The dragged fluid on the body forms the first layer and the following layer of fluid is dragged to the previous layer [15]. As speed increases, viscous *C_d_* seems to decrease, and the dragged fluid on the surface disrupts the body due to the flow turbulence. Thus, the fluid flow turbulence is the one responsible for disrupting the dragged flow sublayers to the object and, as less fluid is dragged to the cyclist, there is less viscous drag [15]. Upon that, pressure drag was the main force responsible for the drag crisis.

This was the first attempt to assess the drag crisis phenomenon on a bicycle–cyclist system. Altogether, the drag crisis phenomenon seems to occur at speeds between 3 m/s and 9 m/s and *Re* is from 3.21 × 10^5^ to 9.63 × 10^5^ in the recruited bicycle–cyclist system. This phenomenon was firstly reported with spheres, and at different ranges of *Re* than what is reported in the present study. With no surprise, the different ranges of *Re* where drag crisis is noted should be related to differences in the bodies´ geometries. Out of the two drag components, pressure *C_d_* is the one explaining the drag crisis; conversely, the role of viscous *C_d_* is minimal. Several studies forecasting the cyclist´s aerodynamics and performance assume *C_d_* as invariant across different speeds [24,25,26,27]. Taking into consideration the drag crisis and how it affects the *C_d_*, it might increase the accuracy of such forecasts in the future. Therefore, end-users, including coaches, cyclists, researchers and support staff should be aware of these *C_d_* variations when running forecasts and models. Altogether, both viscous and pressure drag coefficients contribute to the drag crisis. At lower speeds (<5 m/s), the viscous drag coefficient has higher variation in comparison to the pressure drag coefficient. This might be due to the fluid flow behavior and the disruption of the fluid from the body surface (viscous drag) due to turbulence [15].

The limitations of this research are: (i) The simulations were run in just one position and *C_d_* varies in different positions. Thus, the range of speeds and *Re* at which the drag crisis happens might vary across different positions. (ii) One single elite cyclist was recruited. As such, care should be exercised extrapolating these results to other demographics (e.g., age groups or para-cycling) and competitive levels. In future work it is important to assess the drag crisis phenomenon on different cycling positions. Moreover, a para-cyclist may have different forms/shapes due to the prosthesis used. Upon that, it is important to assess this phenomenon in different geometries and present the *C_d_* variations, wake flow behavior with streamlines, and vorticity at the selected speeds. Moreover, with the intention to simulate different subjects, simulations with higher or lower surface areas might be performed with the same bicycle–cyclist model.

## 5. Conclusions

The *C_d_* variations of a bicycle–cyclist system differ from different object forms. This may suggest that the cyclists’ different adopted positions might have different *C_d_* variations and the drag crisis phenomenon may vary between positions and body forms. In conclusion, a drag crisis phenomenon is noted in a bicycle–cyclist system in the aero position. The crisis happens at speeds from 3 m/s to 9 m/s and *Re* between 3.21 × 10^5^ and 9.63 × 10^5^. The drag crisis is mostly due to a crisis in pressure *C_d_*. It is important to consider extrapolating the results to different competitive levels or cyclists due to different body sizes and measurements.
